# Understanding the dynamics of mastitis in milk yield: Decoding onset and recovery patterns in response to mastitis occurrence

**DOI:** 10.3168/jdsc.2024-0579

**Published:** 2024-07-14

**Authors:** A.L.L. Sguizzato, T.E. da Silva, J.C.C. Chagas, A.M. Argüelo, N.M. Gonçalves, M.I. Marcondes

**Affiliations:** 1 Department of Animal Science, Universidade Federal de Viçosa, Viçosa, Minas Gerais, 36570-900, Brazil; 2 Department of Animal and Veterinary Sciences, University of Vermont, Burlington, VT 05405; 3 Department of Applied Animal Science and Welfare, Swedish University of Agricultural Sciences, Umeå, 90183, Sweden; 4 Uniform-Agri, Assen, Drenthe, 9401LB, the Netherlands; 5 Department of Animal Science, Washington State University, Pullman, WA 99164

## Abstract

No recent study has attempted to model daily milk losses before and after mastitis onset and the moment when it begins. Thus, we aimed to describe the impact of mastitis on milk production based on mastitis level and moment of occurrence. We used data from 11 dairy farms, and the dataset consisted of 885,759 daily individual milk test records from 3,508 cows in different lactations, with an average milk yield (MY) from January 2017 to December 2022. We modeled the impact of mastitis severity (i.e., 1 [mild] and 2 [severe]) based on the drop and recovery of MY following 3 steps. First, we removed milk recorded on the day of diagnosis of mastitis from the dataset and fitted a Wood's curve for each cow and parity. Second, we returned the mastitis data to the dataset and estimated the residual milk loss due to mastitis from 15 d before to 30 d after the mastitis event. Third, we used generalized additive mixed effect models to estimate the residual milk loss, including farm as a random effect. In addition to the random effect of the farm, we also included the predicted milk yield (by Wood's curve) over the influence of mastitis, the day effect before and after mastitis incidence, and the interaction between the predicted value of mastitis and days. On average, mastitis level 2 resulted in a more severe MY drop in all represented stages of lactation (80, 170, and 260 DIM), suggesting a higher loss close to the lactation peak, approximately 130 kg more than mastitis level 1. Moreover, the occurrence of mastitis case level 1 during the early phase of lactation (DIM 80) can cause an average milk loss of 158 L and mastitis level 2, an average loss of 288 L. The estimations suggest that milk drop occurs 14 to 4 d before mastitis onset and can last until 15 to 25 d from the diagnosis, which would be the necessary time for a cow to re-establish their predicted MY. Therefore, our study brings new perspectives to investigate MY drop and recovery due to mastitis infections and how much mastitis can deplete and impair milk production.

## Highlights

• ML 2 caused an average additional loss of 130 kg of milk compared with ML 1.
• ML 1 occurring at 80, 170, and 260 days caused a total loss of 399 kg of milk.
• ML 2 occurring at 80, 170, and 260 days caused a total loss of 710 kg of milk.
• Milk drop occurred 14 to 4 days before mastitis onset.
• To re-establish production, milk drop can last 15 to 25 days from the diagnosis.

## Graphical Abstract

**Figure:**
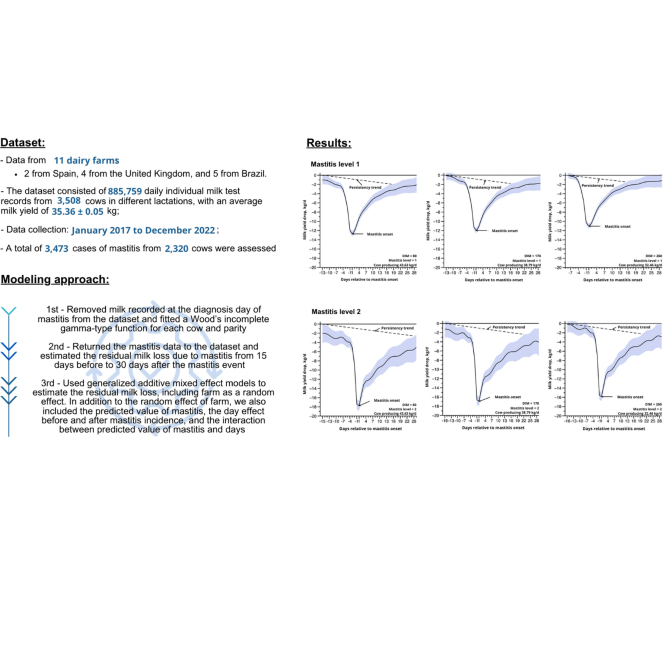
**Summary:** We analyzed data from 11 dairy farms, comprising a dataset of 885,759 daily individual milk test records from January 2017 to December 2022. These records were collected from 3,508 cows in different lactations. The average (± SE) milk yield was 35.36 ± 0.05 kg. A total of 3,473 cases of mastitis from 2,320 cows were assessed. The modeling approach followed that described in the graphical abstract, and mastitis severity was coded as 1 = mild (only gargets were observed during the fore-stripping test) or 2 = severe (symptoms such as redness, inflammation, fever, pus, and blood). The results represent milk yield drop and recovery for mastitis levels (ML) 1 and 2 and indicate a more severe drop for ML 2 in all scenarios.

Mastitis is one of the most common causes of economic losses on dairy farms (Silva et al., 2021) and is known to depress milk yield (**MY**), reduce cow fertility, and increase culling rates (Daros et al., 2019). Moreover, mastitis poses an important welfare concern in dairy operations. In addition to the production losses, dairy cows may undergo discomfort, hunger, and diminished mobility (Nielsen, 2009). Consequently, these multifaceted impacts affect all 3 fundamental aspects of welfare: biological functioning, natural living, and affective state (Fraser et al., 1997).

According to Puerto et al. (2021), the highest reductions in MY were observed during late and mid lactation, accounting for 1,137 and 506 kg of milk, respectively, and resulting in a loss of $710 to $324 of cumulative milk value. van Soest et al. (2016) reported an average annual milk production loss of 336 kg per case per year, equating to approximately $265 per lactating cow. For Heikkilä et al. (2018), clinical mastitis can cause a daily reduction in MY of 1.4 to 3.5 kg, depending on the pathogen.

Milk losses due to mastitis initiate 2 to 4 wk before the diagnosis and can be influenced by previous production, lactation week, and parity (Lescourret and Coulon, 1994; Rajala-Schultz et al., 1999; Nielsen, 2009). Additionally, Nielsen (2009) estimated that, on mastitis day, primiparous cows could lose 5 kg of milk, whereas multiparous cows could have their production reduced from 1 to 8 kg. Moreover, Lescourret and Coulon (1994) and Rajala-Schultz et al. (1999) observed that milk recovery would occur over 2 to 4 wk after the disease identification; however, MY is compromised for the entire lactation. Despite the evidence indicating daily and overall losses during clinical mastitis, recent studies have not attempted to model daily milk losses before the onset of mastitis or after its identification, calling for further investigation into daily milk production re-establishment and recovery time after mastitis identification.

Therefore, given the potential compromise in animal welfare and economic losses associated with mastitis in dairy cows, the focus of this study was to comprehensively elucidate the impact of mastitis on MY. Thus, we aimed to describe the impact of mastitis on milk production based on mastitis level (**ML**) and moment of occurrence. This was achieved through a modeling approach that determined on average the onset and recovery, stratified into 2 levels of severity.

In this retrospective study, we used data from 11 dairy farms (2 from Spain, 4 from the United Kingdom, and 5 from Brazil). The dataset consisted of 885,759 daily individual milk test records from 3,508 cows in different lactations, with an average MY of 35.36 ± 0.05 kg, from January 2017 to December 2022. Milk yield and DIM equal to zero, or any missing data, were removed from the dataset. Additionally, cows should have at least one milk record before 60 DIM and one after 150 DIM to be used in the data analysis and modeling. Thus, a total of 3,473 cases of mastitis from 2,320 cows were assessed. There were 2,456 cases of mild mastitis (1,735 cows) and 1,017 cases of severe mastitis (770 cows), and the number of lactations when mastitis occurred varied from the first to the eleventh lactation. The average prevalence of ML 1 and 2 for the 11 farms studied was 10.9% (minimum = 3.4%; maximum = 16.5%) and 5.7% (minimum = 0.03%; maximum = 13.7%), respectively. Overall, primiparous and multiparous cows had 9.2% and 12.0% of level 1 mastitis prevalence and 4.7% and 5.4% of level 2 mastitis, respectively. For ML 1, the average MY was 34.3 ± 13.6, whereas for ML 2 the average MY was 29.4 ± 15.2 kg/d. The average MY per lactation stage for ML 1 and 2, respectively, were 39.4 ± 13.9 and 34.3 ± 15.6 (from 1 to 80 DIM), 38.8 ± 12.6 and 33.2 ± 15.3 (from 81 to 170 DIM), and 28.9 ± 11.9 and 25.0 ± 13.7 kg/d (from 171 to 260 DIM).

For modeling milk drop and recovery relative to mastitis, days were computed relative to the day when the first clinical sign was observed (from −15 to +30 d), not including zero, and the mastitis day was coded as d 1. We initiated by analyzing the period spanning from 7 d before to 7 d after the onset of mastitis. This involved examining the decline in MY and identifying the onset of decline as well as the duration until full recovery was attained. Initially, our model encompassed a timeframe from −4 to +18 d relative to the mastitis event and, subsequently, we systematically excluded data points within this range and iteratively reran the model to generate new timeframes. This iterative process continued until the removed data points sufficiently replicated the original timeframe following the drop calculation. Mastitis severity was coded as 1 = mild or 2 = severe. The mastitis was considered mild if only gargets (flakes or clots in milk) were observed during the fore-stripping test. If any additional symptoms of mastitis (redness, inflammation, fever, pus, blood, and so on) were observed by the milker, the mastitis was considered a severe case. Although several studies have used 3 grades for mastitis intensity (Wenz et al., 2006; Tomazi et al., 2018; Nagasawa et al., 2019), our preliminary analysis did not result in reasonable estimates when coding 3 levels for mastitis. Therefore, moderate and severe cases were pooled as severe mastitis. It is important to acknowledge that coupling moderate and severe cases of mastitis could cause an overestimation of MY losses for ML 2. Moreover, a mastitis case was considered new if it appeared at least 14 d after the previous or first mastitis case (Tomazi et al., 2018).

We modeled the impact of mastitis based on the drop and recovery of MY following 3 steps. First, we removed milk recorded at the diagnosis day of mastitis (d 1) from the dataset and fitted a Wood's incomplete gamma-type function (Wood, 1967) for each cow and parity,

[1] $$MY_{t}=a\times t^{b}\times exp^{\left(-c\times t\right)},$$

where *MY_t_* = milk yield at the day *t*; *a* = initial milk yield after calving (intercept), and *b* and *c* determine the slope of the curve before and after the peak, respectively.

After running Wood's models for each cow, we added filters for the following coefficients: *a* >0 and *a* <50; *b* >0 and *b* < 1; *c* >0 and *c* <1. The Wood curves were fitted by using the *nlsList()* function from the *nlme* package (R Core Team version 4.2.3).

Second, we returned the mastitis data to the dataset and estimated the residual milk loss (**RML**) due to mastitis from 15 d before to +30 d after the mastitis event:

[2] RML=pMY−MY,

where RML = residual milk loss, pMY = predicted milk yield (by Wood's curve) over the influence of mastitis, and MY = actual milk yield.

Third, to estimate the RML, we used generalized additive mixed effect models (**GAMM**), including farm as a random control effect, through the *gam()* function from the *mgcv* package. In addition to the random effect of farm, we also included the predicted milk yield (by Wood's curve) over the influence of mastitis (**pMY**), the day effect before and after mastitis incidence, and the interaction between pMY and days. We applied cubic regression splines (i.e., bs = “cr”) as smoothing terms and determined the optimal number of knots for the basis functions (i.e., k parameter) by selecting the smallest Akaike information criterion value and checking the adjustment through the *gam.check()* function (where k-index was higher than 1). The REML was chosen as the method for parameter estimation. The model predictions and CI were calculated by using the *predict_gam()* function of the *tidymv* package at 95% confidence.

An overall Wood curve was also fitted for the entire dataset (i.e., without group by cow and parity). The overall curve and adjusted coefficients were used to simulate milk production at 80, 170, and 260 DIM. Also, the persistency was calculated using the general curve based on a linear regression slope from lactation peak to dry-off. The general curve was fitted using the *nls()* (Gauss-Newton algorithm employed as iteration method) function of the *stats* package.

To illustrate the RML during the drop and recovery of mastitis, as an example, we estimated and plotted MY behavior related to the occurrence of mastitis 1 and 2 on d 80, 170, and 260 of lactation (Figure 1, Figure 2). It is essential to observe that the pattern of decline and recovery in milk production occurs in a nonlinear trend. We managed to represent this nonlinearity by the GAMM, which through smoothing functions gives us the flexibility to model such nonlinear patterns (Wood, 2017).

**Figure 1:**
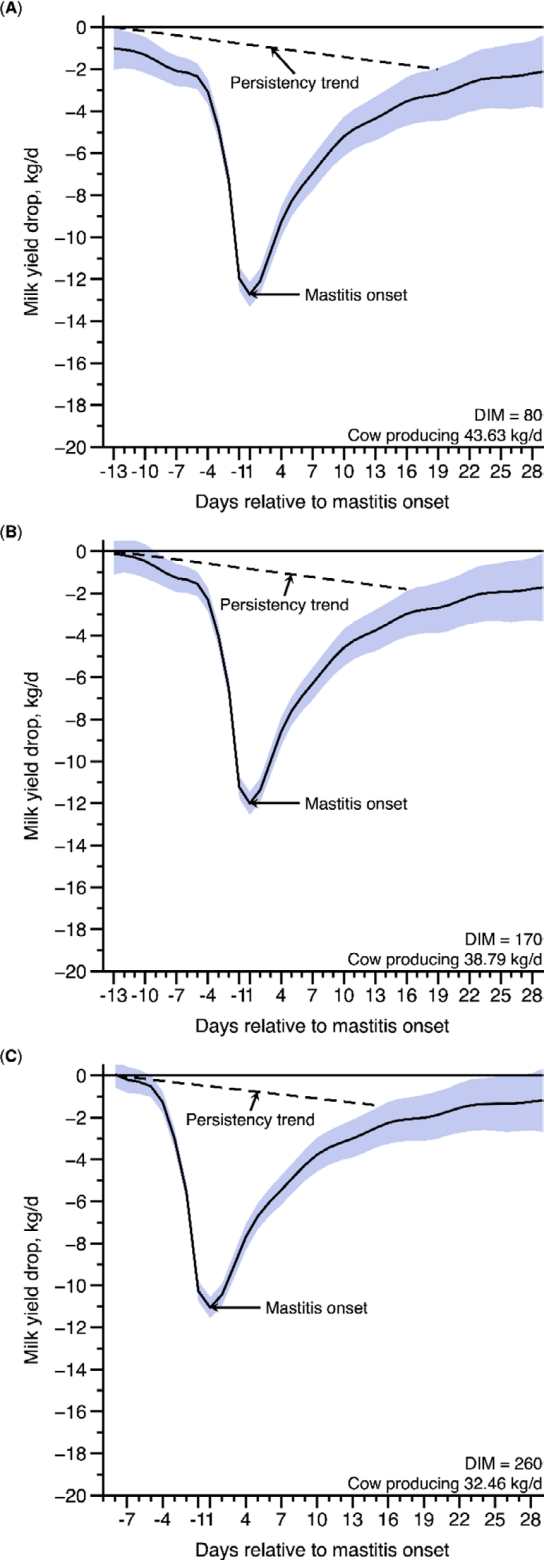
Predicted milk yield drop and recovery (± 95% CI; blue shading) of Holstein cows diagnosed with clinical mastitis level 1 (root mean square error = 8.66 kg/d, and R^2^ = 0.14), in 3 different days of lactation (A: 80 DIM; B: 170 DIM; and C: 260 DIM).

**Figure 2:**
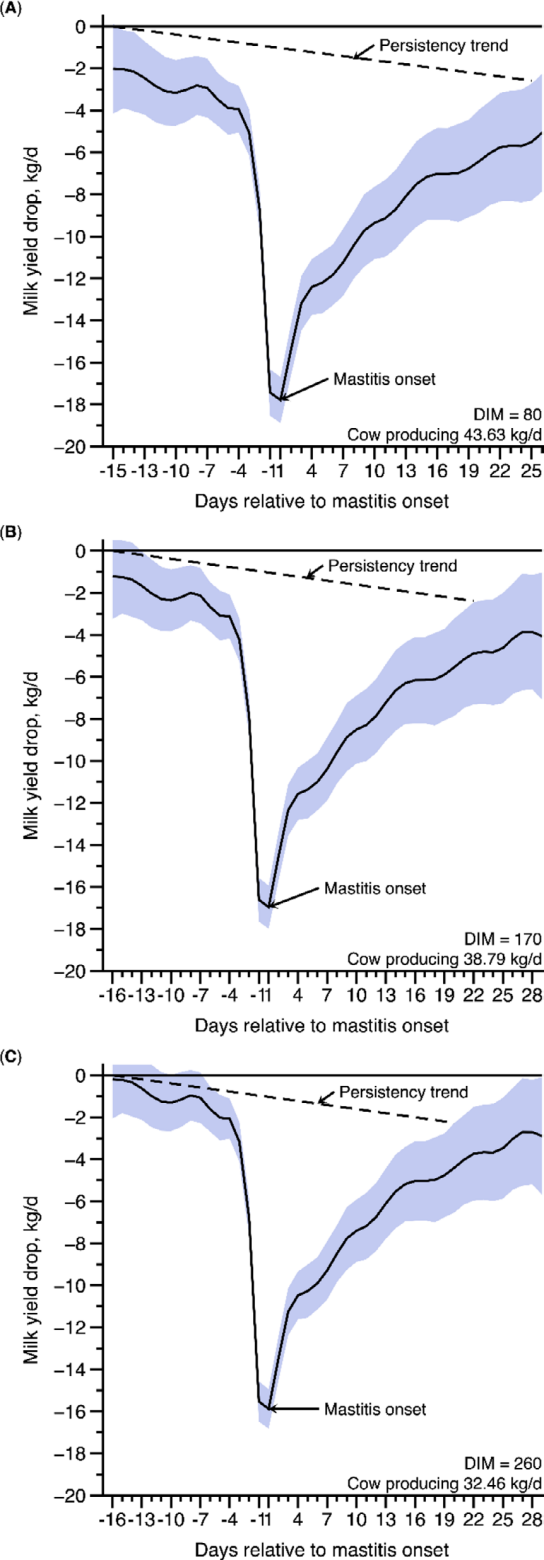
Predicted milk yield drop and recovery (± 95% CI; blue shading) of Holstein cows diagnosed with clinical mastitis level 2 (root mean square error = 10.03 kg/d, and R^2^ = 0.15), in 3 different days of lactation (A: 80 DIM; B: 170 DIM; and C: 260 DIM).

Considering that the average MY of assessed cows was approximately 35.36 kg/d, they were producing 43.63 kg of milk at d 80 of lactation (production peak), 38.79 kg at 170 DIM, and 32.46 kg at 260 DIM. Thus, as can be noticed in Figure 1, at DIM 80, ML 1 would promote a MY drop 12 d before the day of mastitis, and the recovery would occur 19 d after the incidence of this disease. In this specific situation, the total milk loss would account for 158.44 kg of milk, achieving a maximum loss of 12.72 kg of milk/d. Conversely, ML 2, occurring at DIM 80 (Figure 2), would promote a MY drop 14 d before and a recovery 25 d after the mastitis onset, with a total MY loss of 288.46 kg of milk and a peak loss of 17.79 kg/d. In Figure 3, we can observe the average MY per DIM (±CI), fitted general Wood's curve, and the persistency trend after peak (93.55%).

**Figure 3:**
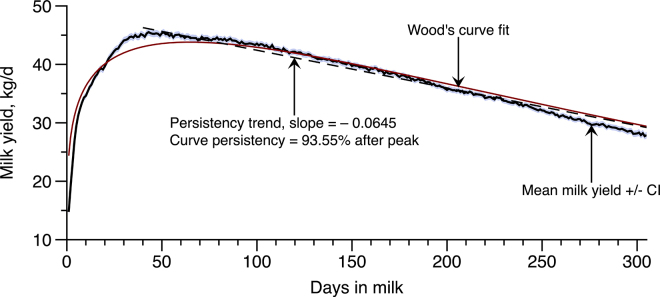
Mean milk yield over the DIM (± 95% CI; black solid line), Wood's curve fit (red solid line), and persistency trend (black dashed line) after the peak of the 3,288 cows. The estimated parameters for the overall Wood's curve were a = 24.4 ± 0.0774, b = 0.184 ± 0.0008, and c = 0.0029 ± 0.00001.

During mid lactation (170 DIM), the first drop in MY would occur 7 d before mastitis incidence and it would prevail for 23 d (recovery 16 d after mastitis diagnosis), depressing 126.15 kg of milk during the entire lactation, for ML 1. However, for ML 2, milk losses would be more prominent, accounting for 237.15 kg of milk and a peak loss of 16.96 kg. In addition, milk drop would begin 11 d before the day of mastitis with a recognized recovery 22 d after it. Last, at the final third of lactation, ML 1 would result in a total loss of 114.51 kg of milk and a peak loss of 11.05 kg, with MY drop and recovery occurring 4 and 15 d before and after mastitis incidence, respectively. Moreover, ML 2 would remain more impactful reducing MY 4 d before mastitis day until 20 d after it, in addition to its ability to reduce 184.39 kg of milk, with a peak loss of 15.88 kg.

On average, ML 2 resulted in a more severe MY drop in all represented stages of lactation (80, 170, and 260 DIM), suggesting a higher loss close to the lactation peak (80 DIM), approximately 130 kg more than ML 1. For the middle and the final lactation stages, the differences between ML 2 and ML 1 would be around 111 and 70 kg, respectively. This pattern of MY loss is expected at lactation peak due to greater MY in this stage. Moreover, ML 1 produced an average daily MY loss of 5 to 6 kg, and ML 2 had an average MY loss of 7 to 7.7 kg, along the lactation states.

At the first third of the lactation curve, both ML 1 and 2 can promote losses from 12 and 14 d, respectively, before the mastitis diagnosis, suggesting that the incidence of this disease can interfere with milk production much earlier than we expected. However, moving on to the last third of the lactation curve, mastitis (ML 1 and 2) can affect MY closer to the diagnosis day (4 d before mastitis onset).

Most of the studies estimating milk losses due to mastitis did not assess when milk losses begin before mastitis onset and how much milk is lost. However, they agree that milk losses can occur 2 to 4 wk before mastitis day and that their recovery can be prolonged until 4 wk after the disease is diagnosed (Lescourret and Coulon, 1994; Rajala-Schultz et al., 1999; Nielsen, 2009), findings similar to the ones observed in our study. A more recent study (Adriaens et al., 2021) assessed MY drop and recovery during perturbations (a perturbation was defined as a period of at least 5 successive days of negative residuals for which the daily MY dropped at least once below 80% of the expected yield). Their average drop and recovery rate were 10 and 11.6 d, respectively, but with some perturbations lasting until 30 d. Moreover, Nielsen (2009) observed that MY loss caused by mastitis could be affected by the severity of the infection, DIM, parity, and production level. Overall, severe cases of mastitis can cause long-term damage to the mammary tissue, and when occurring before lactation peak, clinical mastitis cases are likely to interfere with the differentiation of secretory cells, which would result in yield impairment throughout the entire lactation (Nielsen, 2009). Although our study did not focus on assessing lifetime impairments in milk production, our results correspond to recovery periods close to the ones observed in the literature (Lescourret and Coulon, 1994; Rajala-Schultz et al., 1999), suggesting that these damages can interfere with the entire milk production cycle.

It is noteworthy that our study considers daily losses along the lactation, which can be estimated as a total MY loss. However, while considering the recovery phase, after 15 to 25 d, cows can return to the same pMY at a certain point, considering 93.55% of lactation persistency after peak, as if they did not have a mastitis case. This mathematical estimation may conflict with the biological assumptions of mammary impairment, mainly in the early lactation phase, as mentioned above. Therefore, estimating the recovery rates after a period of severe mastitis infection can be more complex than milk drop itself, considering that long-term biological effects may exist.

According to Santos et al. (2004), mastitis has more severe effects on performance during early lactation. So, as observed in our estimations, a ML 1 occurrence during early phase of lactation (DIM 80) can cause an average milk loss of 158 L and a ML 2, an average loss of 288 L. Østergaard and Gröhn (1999) observed MY losses of 65 kg for primiparous and 117 kg for multiparous cows; however, Østergaard and Gröhn (1999) considered these values underestimated since estimations did not include losses after 5 wk from the mastitis day. Puerto et al. (2021) observed reductions of 1,137 and 506 kg of milk during late and mid lactation, respectively, and van Soest et al. (2016) reported an average milk production loss of cows suffering from clinical mastitis of 336 kg per case per year. Compared with our study, MY losses and days to recover are very distinct. These differences could be explained by the approaches used to estimate MY reduction, by the more intense decrease in MY, and by the recovery rate, which allowed animals to re-establish their production in approximately 2 to 4 wk, as suggested by our data. Moreover, while discussing the potential discrepancy between predicted and actual MY, we might consider whether the observed difference could be greater than initially observed. This speculation arises from the consideration that the Wood's curve, being smoothed over all data points with the use of daily records as input, may mask fluctuations caused by mastitis-induced milk loss. Consequently, it is plausible that the disparity between predicted and actual MY could theoretically be more pronounced than currently observed.

To conclude, the estimations suggest that milk drop occurs 14 to 4 d before mastitis onset and can last until 15 to 25 d from the diagnosis, which would be the necessary time for a cow to re-establish their pMY. In addition, MY loss estimated on mastitis day is greater than the values referenced in the literature and is distinguished between ML. Therefore, our study brings new perspectives to investigate MY drop and recovery due to mastitic infections and how much mastitis can deplete and impair milk production.
